# The influence of thyroid dysfunction on bone metabolism

**DOI:** 10.1186/s13044-014-0012-0

**Published:** 2014-12-20

**Authors:** Dominika Tuchendler, Marek Bolanowski

**Affiliations:** Endocrinology Department, Clinic of Internal Medicine, 4th Military Hospital, Wrocław, Poland; Chair and Department of Endocrinology, Diabetes and Isotope Therapy, Medical University Wrocław, Wrocław, Poland

**Keywords:** Thyroid hormones, Thyroid diseases, Osteoporosis, Fractures

## Abstract

Loss of bone mineral density due to osteoporosis is the main cause of fragility fractures and leads to dropped quality of life and increased mortality. Disturbance of balance between bone formation and bone resorption is dangerous, can cause loss of bone mass and disruption of it’s architecture. Correct development, achievement of peak bone mass and normal functioning of human skeleton depend on different factors. The pivotal role in bone metabolism play thyroid hormones. Both excess as well as deficiency of fT_4_ and fT_3_ can be potentially deleterious for bone tissue.

The aim of this study is to review the current literature concerning the role of thyroid hormones on bone metabolism.

## Introduction

A lot of different factors are necessary to correct and harmonious development as well as normal functioning of skeleton: genetic conditions, hormonal and metabolic homeostasis, balanced diet, mechanical load [1]. Any disturbances of those agents can lead to serious and dangerous consequences like length reduction, deformations, fractures. Their results depend, between other, on one’s age, type of disorder and its duration. We don’t have any influence on some of mentioned before factors, but part from them is possible to modify. It is very significant because abnormalities and irregularities of bone mineralization can lead to loss of bone density and to osteoporosis in final effect [1].

Because of rising number of newly diagnosed osteoporosis cases it is necessary to perform an active investigation for all potential secondary conditions which can lead to reduced bone mineral density. It is very important because osteoporotic fractures are directly related with dropped quality of life, disability and mortality. It is estimated that 40% of women and 13-22% of men aged about 50 will experience to the end of life at least one fracture connected with osteoporosis – mainly proximal femur, spine or forearm [2]. Society’s aging causes that the number of people with osteoporotic fracture in Poland will amount in year 2025 about 3.23 millions. It means growth about 32.8% relatively to year 2008. Besides, unfortunately a trend appeared that osteoporosis, mainly secondary, is diagnosed in more and more younger age groups.

There is a lot of endocrinological reasons of secondary osteoporosis (for example: Cushing's syndrome, hyperparathyroidism, hypogonadism, acromegaly, diabetes mellitus etc.) [3]. It is necessary to remember about them because their negative influence on bone tissue is potentially reversible. In this group also thyroid gland hormonal disorders play an important role. Human interest about the influence of thyroid hormone on skeleton lasts since long time. Von Recklinghausen described a correlation between numerous fractures and state of hyperthyroidism over 120 years ago [4]. Researches and discussions persist if the key role on bone tissue has TSH or thyroid hormones? Is this question important? The answer is yes, because disorders of thyroid gland and disturbances of axis TRH-TSH-fT3 and fT4 are very often; concern millions of people in global scale. The implications for bone leading to bone loss causes that willingness of the answer to the question about the influence of thyroid dysfunction on bone is quite great.

## Review

### Molecular actions of thyroid hormones in bone tissue

The action of thyroid hormones is mediated by thyroid hormone receptors (TR), which are encoded by THRA and THRB genes [4]. Each of TR owns a few subtypes. We can differ TRα1, TRα2, TRβ1, TRβ2 receptors [5-7]. They are localized not only on thyrocytes, but also on majority human tissues and cells. Only TRβ2 is connected with hypothalamus and pituitary, where inhibits the secretion of TRH and TSH [8,9]. Their expression in bone directly means that this tissue is under the influence of thyroid hormones. Osteoblasts and chondrocytes exert the expression of both TRα and TRβ [5,6,10], but concentration of TRα1 is ten times larger than TRβ1 [4,5]. TRα1 is considered as a main functional mediator of triiodothyronine in skeleton [5,11,12]. The biological role of TRα2 is unknown [13]. Deficiency or dysfunction of TRα leads to growth retardation, delayed bone age, perturbations in bone mineralisation and decreased BMD [8,14]. T3 regulates the chondrogenesis and bone mineralisation [6]. T3 stimulates the IL-6 and IL-8, intensifies the effects of IL-1 and IL-6, augments the synthesis of osteocalcin, collagen type 1, increases proliferation, differentiation and apoptosis of osteoblasts [8].

The receptors for TSH (TSHR) are located not only in thyroid follicular cells, but also in osteoblasts and osteoclasts [4,5]. Data from scientific reports indicates that TSH is considered as a negative regulator of bone turnover [4]. Its direct action on bone tissue cells leads to enhanced bone remodeling and osteoporosis [15,16].

### The influence of overt hyperthyroidism on bone

Thyroid hormones play pivotal role in linear development of skeleton. They are necessary to achieve peak bone mass [8]. But the excess of thyroid hormones in childhood can lead to premature accretion of growth plates and cranial sutures and finally short stature and craniosynostosis [8,13,17,19]. In adults, overt hyperthyroidism leads to acceleration of bone turnover and loss of mineral density in 10-20%, mainly in cortical bone [8,13,19]. The cycle of bone remodeling is shortened in almost 50% (from 200 to 113 days), and the proportions between bone formation and bone resorption are disturbed [20]. The phase of bone formation is reduced in 2/3, which effects in loss of over 10% mineralized bone on one cycle [21]. In consequence thyrotoxicosis leads to increased risk of fractures [8,13,20,22]. Elevated serum concentrations of IL-6 are observed in people with hyperthyroidism [23,24]. IL-6 stimulates the production of osteoclasts and may be a mediator of parathyroid hormone on bone tissue [23,24]. Adverse changes in bone metabolism seen in hyperthyroidism are connected with negative calcium balance, hypercalcaemia and hypercalciuria [13,23].

Since times of von Recklinghausen many authors returned in their researches to the topic of thyroid gland hormonal dysfunction and its influence on skeleton (18). Svare et al. in cross-sectional, population-based study HUNT 2 in Norway exerted that women with lowest TSH concentrations (<0.5 m U/l) had lower forearm BMD than reference category. In this research also the prevalence of osteoporosis was higher in women with history of hyperthyroidism [25]. Vestergaard et al. in a meta-analysis from year 2003 noted that BMD was significantly decreased in patients with untreated hyperthyroidism. During anti-thyroid treatment a statistically significant increase in BMD was observed and it reversed to initial normal levels with even temporary increase above normal levels within 1 to 4 years after diagnosis. The risk of hip fractures was correlated positively with age at time of diagnosis of hyperthyroidism [26]. Ercolano et al. examined 57 women with diagnosed Graves’ disease, who were in state of euthyroidism (for minimum 6 months) during study, but with previous history of hyperthyroidism and wanted to determine the correlation with BMD and TSH receptor antibodies (TRAb). They concluded that BMD was decreased only in postmenopausal women and they found a negative correlation between Z-score of lumbar spine and TRAb [27]. Tuchendler et al. in their study on 38 premenopausal women with newly diagnosed hyperthyroidism noted that a decrease in bone mineral density occurred in cortical bone (femoral neck). After one year of anti-thyroid treatment an increase in BMD was observed, but it was still lower that in control group, which indicated that correcting the hormonal dysfunction didn’t fully normalize bone density. The negative impact on bone equilibrium expressed first as an increase in markers of bone turnover. This observation permitted detection of bone loss at an earlier stage preceding changes observed in X-ray densitometry. After one year of anti-thyroid treatment concentrations of bone turnover markers decreased in a statistically significant way [28]. El Hadidy et al. found out that not only women, but also men suffering from hyperthyroidism (Graves’ disease or multinodular toxic goiter) had significant bone loss and it was related with the severity and duration of thyrotoxic state [29].

Not only current thyrotoxicosis is a risk factor of fragility fracture, but also previous history of hyperthyroidism belongs to independent risk factors for hip and vertebral fractures. Wejda et al. described that hyperthyroidism was found 2.5-fold more often in hip fracture patients than in controls [30]. Bauer et al. noted that postmenopausal women with decreased TSH concentrations (<0.1 mU/L) had a threefold increased risk for hip and a fourfold increased risk for vertebral fracture compared with women with normal TSH levels (from 0.5 to 5.5 mU/L). There was an interesting detail that a history of hyperthyroidism was also associated with a twofold increase in hip fracture [31]. It can mean indirectly that BMD doesn’t return to normal ranges after anti-thyroid treatment.

Vestergaard et al. in their case–control study in the year 2000 in Denmark noticed an increase in the risk of any fracture within the first 5 years after a diagnosis of hyperthyroidism. Use of anti-thyroid drugs was associated with a significantly reduced fracture risk independently of the dose used [32]. In this aspect another study of the same author was very interesting because he observed an increased fracture risk in hyperthyroidism treated with radioactive iodine alone, but not in subjects that had received both radioactive iodine and methimazole or other types of anti-thyroid therapy. His study considered of 864 patients with diffuse toxic goiter or toxic nodular goiter [33].

### The influence of subclinical hyperthyroidism on bone

We have very little data from medical studies describing the influence of subclinical hyperthyroidism on bone metabolism. It is an object of discussion, still. Lee et al. concluded that women having subclinical hyperthyroidism had reduced femoral neck BMD but not lumbar spine BMD. Serum concentration of bone turnover markers showed no differences between studied and control group [34]. Folder et al. in his cross-sectional study indicated that the bone mineral density of the lumbar spine, femoral neck and the midshaft of the radius were not significantly decreased in premenopausal patients with endogenous subclinical hyperthyroidism but he also concluded that long-lasting endogenous subclinical hyperthyroidism may be a contributing factor to the development of osteoporosis in some postmenopausal women, mostly at sites where cortical bone preponderates [35]. Vadiveloo et al. wanted to investigate the long-term outcomes, between other fracture risk, for patients with endogenous subclinical hyperthyroidism (SH). They concluded that patients with endogenous SH have an increased risk of fracture with a hazard ratio 1.25, but when they developed overt hyperthyroidism or euthyroidism and were excluded from the study, this association was lost [36]. Lee et al. concluded that older men (>65 years old) with subclinical hyperthyroidism were at increased risk for hip fracture. No clear association between subclinical dysfunction and fracture was observed in women in this study [37].

The situation is complicated with reference to premenopausal women. Ugur-Altun et al. didn’t find any difference in BMD between group of premenopausal women with untreated subclinical hyperthyroid in the course of Graves’ disease (age 33 ± 5 years) and healthy women (age 35 ± 3 years) [38]. Tauchmanovà et al. examined the group of 60 patients with endogenous subclinical hyperthyroidism due to multinodular goiter: 30 of women were premenopausal and 30 early postmenopausal (mean age, 40.9 ± 7.3 and 57.7 ± 6.75, respectively). She observed a significant decrease in femoral BMD both in pre- and postmenopausal patients compared to controls (but being greater in those postmenopausal). Also lumbar BMD was decreased but only in postmenopausal patients [39]. Rosario noticed a significant increase in serum markers of bone formation and resorption in premenopausal women with subclinical hyperthyroidism aged < 65 years. In his research bone mineral density was lower in the femoral neck but not in lumbar spine in patients before menopause, whereas lower BMD at both sites was observed in postmenopausal patients [40].

### The influence of overt hypothyroidism on bone

Untreated hypothyroidism in childhood leads to growth retardation or even growth arrest, disturbances of endochondral ossification, delayed bone age and persistent short stature [8,13,19]. Hypothyroidism causes general hypometabolism [20]. Bone formation processes are slowed in 50%, bone resorption processes – in 40% [20]. The calciuria is reduced, serum concentration of osteocalcin and alkaline phosphatase is decreased, but serum concentration of parathyroid hormone and vitamin D can be elevated [20]. The influence of deficiency of thyroid hormones on bone metabolism is discussed. Nevertheless, it is considered that hypothyroidism is related with increased risk of fractures, although their mechanism remains unclear [9]. Hanna et al. noted that there was no evidence for a difference in bone mineral density in patients receiving replacement doses of thyroxine irrespective of the etiology of hypothyroidism [41]. Leger et at proved that LT_4_ replacement therapy among children with congenital hypothyroidism is not detrimental to the skeletal mineralization [42].

Vestergaard et al. noted that there was a temporary increase in fracture risk within the first 2 years after diagnosis of primary idiopathic hypothyroidism. The fracture risk was mainly increased in the age group above 50 years, and the increased risk was limited to the forearms [43]. In another study of the same author was noticed that there was an increase in the risk of any fracture within the first 10 years after a diagnosis of hypothyroidism. No effect of levothyroxine on fracture risk was present in that research [32]. Gonzalez-Rodriguez et al. did not find any association between hypothyroidism and decreased bone mineral density or vertebral and non-vertebral fractures among 400 women suffering from hypothyroidism [44]. Lee et al. concluded that men over 65 years old with subclinical hypothyroidism were at increased risk for hip fracture. Whether treatment of the subclinical syndrome reduced this risk was unknown [38].

Polovina et al. noticed that postmenopausal patients with subclinical hypothyroidism, in particular of autoimmune origin, have higher FRAX scores and a thus greater risk for low-trauma hip fracture than euthyroid postmenopausal women [45]. Tuchendler et al. noted that newly diagnosed hypothyroidism among premenopausal women (average age 33,37 ± 10,83) did not have an influence on bone density [28].

### The influence of suppressive doses of levothyroxine on bone

Suppressive doses of L-T_4_ are used in patients with differentiated thyroid carcinoma as a complementary treatment after thyroidectomy and radioactive iodine therapy. Although an endogenous hyperthyroidism is a risk factor of secondary osteoporosis, the effects supraphysiological doses of levothyroxine on bone are still under discussion. Heemstra et al. suggested that in postmenopausal women after long-term suppressive thyroxine replacement therapy decreased BMD and increased risk of osteoporosis was observed, whereas it wasn’t noted among men and premenopausal women [46]. Quan et al. analyzed 11 researches concerning the effects of suppressive thyroxine therapy in patients with well-differentiated thyroid cancer on bone metabolism. They didn’t notice significant change in bone mineral density among premenopausal women and men. Findings for postmenopausal women remained unclear and needed further studies [47].

Lee et al. observed 94 females (mean age, 50.84 ± 11.43 years) receiving L-T4 after total or near total thyroidectomy and radioactive iodine therapy for thyroid cancer. They detected no significant decrease in BMD or bone turnover markers according to TSH level or free T_4_ level. Also, the prevalence of osteoporosis and osteopenia was not increased in this study [48]. Franklyn et al. concluded that despite long-term thyroxine therapy (mean duration 7.9 [range 1–19] years) which resulted in higher serum thyroxine and lower serum TSH concentrations than in the controls, the patients showed no evidence of lower bone mineral density than the controls at any site. There was a decrease in bone density with age in both groups. They suggested that thyroxine alone didn’t have a significant effect on bone mineral density and hence on risk of osteoporotic fractures [49].

Kung et al. observed for 2 years 46 postmenopausal women with carcinoma of thyroid gland to evaluate the rate of bone loss. All patients were receiving a stable dose of L-T_4_ (170 ± 60 micrograms/day or 3.0 ± 1.4 micrograms/kilogram/day) for more than 1 year. They noticed that T4-suppressive therapy was associated with bone loss in postmenopausal women [50]. Uzzan et al. performed a meta-analysis (by pooling standardized differences, using a fixed effect model) of all published controlled cross-sectional studies (41, including about 1250 patients) concerning the impact of thyroid hormones therapy on bone mineral density. They found out that for lumbar spine and hip (as for all other sites), suppressive thyroid hormones therapy was associated with significant bone loss in postmenopausal women but not in premenopausal women [51].

There is no data in literature evaluating the fracture risk and the concentrations of bone turnover markers in premenopausal women treated with suppressive doses of levothyroxine by the reason of differentiated thyroid carcinoma.

## Conclusions

Thyroid hormones are necessary to normal development and function of human skeleton. This is evidently visible in conditions of hyper- and hypothyroidism. Although it is still unclear if bone changes observed in state of thyrotoxicosis are related to lack of TSH or to excess of thyroid hormones or both of them. Overt hyperthyroidism leads to decreased BMD and increased fracture risk. Subclinical hyperthyroidism also causes a small reduction in BMD and increased risk of fracture but only in men ant postmenopausal women. Then suppressive doses of T_4_ can contribute to reduced BMD due to patient’s sex and gender (in postmenopausal females). Hypothyroidism has controversial influence on bone metabolism but probably leads to increased fracture risk.

## Abbreviations

BMD: Bone mineral density
SH: Subclinical hyperthyroidism
TR: Thyroid hormone receptor
TRAb: TSH receptor antibodies
TSHR: Receptor for TSH
